# Supplementary material for: Resolving the full spectrum of human genome variation using Linked-Reads
Source: Genome Res. 2019 Apr;29(4):635–45. doi: 10.1101/gr.234443.118 (PMC6442396; doi:10.1101/gr.234443.118)
Supplement: Supplemental Material [file supp_gr.234443.118_Supplemental_longranger-2.2.zip › longranger-2.2.tar.bz2!/lr2.2-public/lib/stan/cmdstan/stan/doc/api/html/functions_func_c.html]

Stan: Class Members - Functions


|  |  |
| --- | --- |
|  | Stan  2.5.0  probability, sampling & optimization |


- Main Page
- Related Pages
- Namespaces
- Classes
- Files

- Class List
- Class Index
- Class Hierarchy
- Class Members

- All
- Functions
- Variables
- Typedefs
- Enumerator
- Related Functions

- a
- b
- c
- d
- e
- f
- g
- h
- i
- j
- k
- l
- m
- n
- o
- p
- q
- r
- s
- t
- u
- v
- w
- x
- z
- ~


All Classes Namespaces Files Functions Variables Typedefs Enumerations Enumerator Friends Macros Pages

### - c -

- called()
  : stan::common::recorder::sum\_values
- central\_interval()
  : stan::mcmc::chains< RNG >
- chain()
  : stan::agrad::chainable
  , stan::agrad::partials\_vari
  , stan::agrad::precomp\_v\_vari
  , stan::agrad::precomputed\_gradients\_vari
  , stan::agrad::stored\_gradient\_vari
- chainable()
  : stan::agrad::chainable
- chainable\_alloc()
  : stan::agrad::chainable\_alloc
- chains()
  : stan::mcmc::chains< RNG >
- check()
  : stan::math::detail::bounded< T\_y, T\_low, T\_high, T\_result, y\_is\_vec >
  , stan::math::detail::bounded< T\_y, T\_low, T\_high, T\_result, true >
- cholesky\_corr()
  : stan::io::reader< T >
- cholesky\_corr\_constrain()
  : stan::io::reader< T >
- cholesky\_corr\_unconstrain()
  : stan::io::writer< T >
- cholesky\_corr\_var\_decl()
  : stan::gm::cholesky\_corr\_var\_decl
- cholesky\_factor()
  : stan::io::reader< T >
- cholesky\_factor\_constrain()
  : stan::io::reader< T >
- cholesky\_factor\_unconstrain()
  : stan::io::writer< T >
- cholesky\_factor\_var\_decl()
  : stan::gm::cholesky\_factor\_var\_decl
- cmd\_line()
  : stan::io::cmd\_line
- cols()
  : stan::math::LDLT\_factor< stan::agrad::var, R, C >
  , stan::math::LDLT\_factor< T, R, C >
- comma()
  : stan::io::csv\_writer
- command()
  : stan::io::cmd\_line
- complete\_adaptation()
  : stan::mcmc::stepsize\_adaptation
- compute()
  : stan::agrad::LDLT\_alloc< R, C >
  , stan::math::LDLT\_factor< stan::agrad::var, R, C >
  , stan::math::LDLT\_factor< T, R, C >
- compute\_criterion()
  : stan::mcmc::base\_nuts< M, P, H, I, BaseRNG >
  , stan::mcmc::dense\_e\_nuts< M, BaseRNG >
  , stan::mcmc::diag\_e\_nuts< M, BaseRNG >
  , stan::mcmc::unit\_e\_nuts< M, BaseRNG >
- compute\_indent()
  : stan::gm::argument
- compute\_next\_window()
  : stan::mcmc::windowed\_adaptation
- conditional\_statement()
  : stan::gm::conditional\_statement
- constrained\_param\_names\_visgen()
  : stan::gm::constrained\_param\_names\_visgen
- cont\_params()
  : stan::mcmc::sample
- contains()
  : validate\_identifier
- contains\_i()
  : stan::io::dump
  , stan::io::var\_context
  , stan::json::json\_data
- contains\_nonparam\_var()
  : stan::gm::contains\_nonparam\_var
- contains\_r()
  : stan::io::dump
  , stan::io::var\_context
  , stan::json::json\_data
- contains\_var()
  : stan::gm::contains\_var
- ConvergenceOptions()
  : stan::optimization::ConvergenceOptions< Scalar >
- convert\_offset\_rtl\_2\_ltr()
  : stan::json::json\_data\_handler
- corr()
  : stan::io::reader< T >
- corr\_constrain()
  : stan::io::reader< T >
- corr\_matrix()
  : stan::io::reader< T >
- corr\_matrix\_constrain()
  : stan::io::reader< T >
- corr\_matrix\_unconstrain()
  : stan::io::writer< T >
- corr\_matrix\_var\_decl()
  : stan::gm::corr\_matrix\_var\_decl
- corr\_unconstrain()
  : stan::io::writer< T >
- correlation()
  : stan::mcmc::chains< RNG >
- coupled\_ode\_observer()
  : stan::math::coupled\_ode\_observer
- coupled\_ode\_system()
  : stan::math::coupled\_ode\_system< F, double, double >
  , stan::math::coupled\_ode\_system< F, double, stan::agrad::var >
  , stan::math::coupled\_ode\_system< F, stan::agrad::var, double >
  , stan::math::coupled\_ode\_system< F, stan::agrad::var, stan::agrad::var >
- cov\_matrix()
  : stan::io::reader< T >
- cov\_matrix\_constrain()
  : stan::io::reader< T >
- cov\_matrix\_unconstrain()
  : stan::io::writer< T >
- cov\_matrix\_var\_decl()
  : stan::gm::cov\_matrix\_var\_decl
- covar\_adaptation()
  : stan::mcmc::covar\_adaptation
- covariance()
  : stan::mcmc::chains< RNG >
- csv()
  : stan::common::recorder::csv
- csv\_writer()
  : stan::io::csv\_writer
- curr\_f()
  : stan::optimization::BFGSMinimizer< FunctorType, QNUpdateType, Scalar, DimAtCompile >
- curr\_g()
  : stan::optimization::BFGSMinimizer< FunctorType, QNUpdateType, Scalar, DimAtCompile >
- curr\_p()
  : stan::optimization::BFGSMinimizer< FunctorType, QNUpdateType, Scalar, DimAtCompile >
- curr\_x()
  : stan::optimization::BFGSMinimizer< FunctorType, QNUpdateType, Scalar, DimAtCompile >


---

[ Stan Home Page ]

*© 2011–2014,
Stan Development Team.*
